# Phase-specific dynamics of coagulation factors V, VIII, and XIII during liver transplantation: insights from a prospective study

**DOI:** 10.1186/s13741-026-00695-0

**Published:** 2026-05-06

**Authors:** Sarah Thaler, Anna Zorn, Isabell Aster, Dionysios Koliogiannis, Markus Guba, Bernhard W. Renz, Philipp Groene

**Affiliations:** 1 Department of Anaesthesiology, LMU University Hospital, LMU Munich, Marchioninistr. 15, Munich, 81377 Germany; 2 Department of General, Visceral and Transplantation Surgery, LMU University Hospital, LMU Munich, Munich, Germany

**Keywords:** Liver transplantation, Hemostasis, Coagulation management, Factor V (FV), Factor VIII (FVIII), Factor XIII (FXIII), Fibrinolysis, Perioperative monitoring

## Abstract

**Background:**

Liver dysfunction is associated with a rebalanced but fragile hemostatic system, in which alterations in coagulation factor synthesis and activity contribute to both bleeding and thrombotic risks. During liver transplantation, this fragile equilibrium is further challenged by major physiological stress. This study aimed to characterize the phase-specific intraoperative dynamics of coagulation factors V, VIII, and XIII (FV, FVIII, and FXIII) to better understand hemostatic modulation and its clinical implications during liver transplantation.

**Methods:**

A subset of 17 liver transplant recipients receiving transfusion support without administration of recombinant FVIII or FXIII was analyzed. Measurements were obtained at three defined intraoperative time points: T1 anesthesia induction, T2 end of anhepatic phase, T3 end of surgery. Activities of FV, FVIII, and FXIII were quantified and analyzed for temporal trends.

**Results:**

All three coagulation factors declined during surgery. FV was already markedly reduced at T1 (37% (22/55)) and further decreased to 26% at T3 ((19/34); p = 0.0309). FVIII showed supranormal levels at T1 (193% (160/254) and declined to near-normal levels at T3 (109% ((67/143); p < 0.0001). FXIII remained close to the lower limit of normal (T1: 68% (50/85)); T3: (63% (55/78)) without significant change.

**Conclusion:**

This prospective analysis reveals distinct, phase-specific trajectories of FV, FVIII, and FXIII during liver transplantation. Understanding these differential patterns may help identify critical periods of hemostatic vulnerability and guide individualized factor-specific therapeutic interventions to optimize perioperative coagulation management in liver transplant recipients.

**Trial registration:**

German Clinical Trials Register (DRKS00032827).

## Background

The liver is central to the hemostatic balance, synthesizing the majority of both procoagulant and anticoagulant factors. These include key components of secondary hemostasis such as fibrinogen, prothrombin, and factors V, VII, IX, and X, as well as natural anticoagulants like antithrombin, protein C, and protein S. In liver dysfunction, this finely tuned equilibrium can be profoundly disrupted. The resulting hemostatic state is not merely hypo- or hypercoagulable, but represents a complex and dynamic rebalancing of coagulation. This rebalanced state is inherently fragile and susceptible to perturbations, rendering liver patients vulnerable to both bleeding and thrombotic complications (Tripodi and Mannucci 2011; Forkin et al. 2018; Northup et al. 2008; Henry and Northup 2017; Tripodi et al. 2010).

While hepatic synthesis is crucial for hemostasis, individual coagulation factors differ in their cellular origins. Factor V (FV) is almost exclusively produced by hepatocytes in the liver, whereas factor VIII (FVIII) and factor XIII (FXIII) synthesis occurs both hepatically and extrahepatically. Notably, a substantial proportion of FXIII originates from myeloid cells, including megakaryocytes and macrophages (Tripodi and Mannucci 2011; Rosing and Tans 1997; Duga et al. 2004; Hollestelle et al. 2004; Hollestelle et al. 2001 Sep; Sinegre et al. 2018; Muszbek et al. 2011; Himmelreich et al. 1993). Understanding the timing and magnitude of factor fluctuations is critical for optimizing hemostatic management in patients at high risk of bleeding or thrombosis.

Liver transplantation is the only definitive treatment in end-stage liver failure. However, beyond the pre-existing coagulopathy intrinsic to liver failure, the procedure itself imposes additional physiological stress that can induce unpredictable alterations in the tenuous hemostatic balance (Hartmann et al. 2016; Thai et al. 2020). Surgical trauma, ischemia–reperfusion injury, and immunologic responses create a highly dynamic coagulation environment manifesting in fluctuations in plasma levels and activities of coagulation factors. The diversity of cellular sources involved in the synthesis of coagulation factors adds an additional layer of complexity with potential implications for the hemostatic management.

In a subanalysis of a larger prospective study on hyperfibrinolysis during liver transplantation, intraoperative trajectories of coagulation factors V, VIII, and XIII were systematically investigated in patients who received transfusion support reflecting the clinical reality of most transplant cases (Thaler et al. 2025). These factors were selected to capture complementary aspects of perioperative hemostasis: FV as a marker of hepatocellular synthetic capacity due to its predominant hepatic origin and short half-life; FVIII as an indicator of endothelial activation and rebalanced hemostasis; and FXIII as a key determinant of fibrin cross-linking and clot stability, particularly relevant in the context of bleeding and transplantation-associated hyperfibrinolysis. Given their distinct cellular origins and physiological functions, this study aimed to delineate phase-dependent fluctuations across key surgical stages and to explore their relationship with concurrent fibrinolytic profiles. While previous studies have focused on isolated time points in transfusion-free liver transplantation (Rengeiné et al. 2020), this analysis provides an integrated, dynamic view of phase-specific hemostatic shifts throughout the transplantation process. Such characterization may enhance the understanding of hemostatic modulation during liver transplantation and help identify critical periods of vulnerability to guide targeted therapeutic interventions.

## Methods

This study was a prospective subanalysis embedded within an observational cohort study investigating coagulation dynamics in liver transplantation. The study protocol was approved by the ethics committee of Ludwig-Maximilians-University (LMU), Munich, Germany (No 23—0613; 2023, Aug 30) and conducted in accordance with the Declaration of Helsinki. Written informed consent was obtained from all patients before study inclusion. Exclusion criteria comprised age < 18 years and refusal to participate. The study was registered at the German Clinical Trials Register (DRKS00032827) prior to enrolment.

For the current analysis, a subset of 17 patients was selected from the overall cohort of 30 liver transplant recipients enrolled between September 2023 and June 2024. Patients who received recombinant factor VIII or factor XIII concentrates during transplantation were excluded. Administration of Fresh Frozen Plasma (FFP) did not lead to exclusion since FFP contains all coagulation factors but at standard clinical doses only slightly compensates for individual factor deficiencies without inducing a procoagulant state (Kozek-Langenecker et al. 2011; Rassi et al. 2020). Patients undergoing retransplantation or with missing values at any predefined sampling time points were also excluded to ensure data completeness and consistency.

Blood samples were collected in citrate and EDTA tubes at predefined perioperative time points corresponding to key procedural phases: T1 induction of general anesthesia, T2 end of anhepatic phase, T3 end of surgery. Activities of coagulation factor V (Thromborel® S), factor VIII (chromogenic assay), and factor XIII (enzymatic chromogenic assay) were measured in the LMU Department of Laboratory Medicine together with routine coagulation parameters such as platelet count, Quick/INR, and aPTT.

### Statistics

Statistical analysis was performed using GraphPad Prism 10 (GraphPad Software, San Diego, CA, USA). Differences across time points were evaluated using either repeated measure one-way ANOVA or Friedman’s test, depending on data distribution determined by prior normality testing. Post-hoc correction for multiple comparisons was applied using the Holm-Sidak method (ANOVA) or Dunn’s test (Friedman test). Spearman’s rank correlation coefficient was used to assess correlations between continuous variables. A two-tailed *p* < 0.05 was considered statistically significant.

## Results

Descriptive statistics for the study cohort including demographics, etiology and classification of liver disease, preoperative hemostatic profile, surgical data and postoperative complications are summarized in Table 1.

**Table 1:** Demographics and surgery data

Demographics of patients and surgery data (*n* = 17)	
Age (years)	58 (52/63)
Sex (male/female)	11 (65%)/6 (35%)
BMI (kg/m^2^)	24 (21/30)
Arterial hypertension	8 (47%)
Diabetes mellitus Type II	6 (35%)
NASH	1 (6%)
Etiology of liver pathology	
Alcoholic liver disease	5 (29%)
Biliary cirrhosis	5 (29%)
Autoimmune hepatitis	2 (12%)
HCC	2 (12%)
Others	3 (18%)
Child–Pugh-Score (*n* = 15)	
Child A	2 (12%)
Child B	7 (41%)
Child C	6 (35%)
MELD-Score	19 (13/27)
Preoperative hemostatic profile	
Platelet count (G/l)	63 (49/99)
Quick (%)	50 (35/57)
INR	1.5 (1.4/2.0)
Fibrinogen (mg/dl)	155 (130/186)
Length of surgery (min)	295 (242/334)
Blood loss (ml)	3300 (1700/5430)
Red Blood Cell concentrate (ml)	750 (500/1250)
Autologous blood transfusion (ml)	580 (142/954)
Platelet concentrate (ml)	0 (0/600)
Fresh Frozen Plasma (ml)	1500 (1125/2250)
Fibrinogen (g)	4 (0/8)
Prothrombin complex concentrate (I.E.)	0 (0/2050)
Postoperative complications (up to 7 days post transplantation)	
Thromboembolic events	3 (18%)
Primary Graft Dysfunction	1 (6%)
Bleeding complications	0

Values are presented as median with IQR or number (percentage), as appropriate. Preoperative hemostatic parameters comprised platelet count, Quick/INR and fibrinogen level

*BMI* Body Mass Index, *HCC* Hepatocellular carcinoma, *MELD* Model for End stage Liver Disease, *NASH* Nonalcoholic Steatohepatitis

Data are presented as median with interquartile range (IQR, Q1/Q3).

**Fig. 1:**
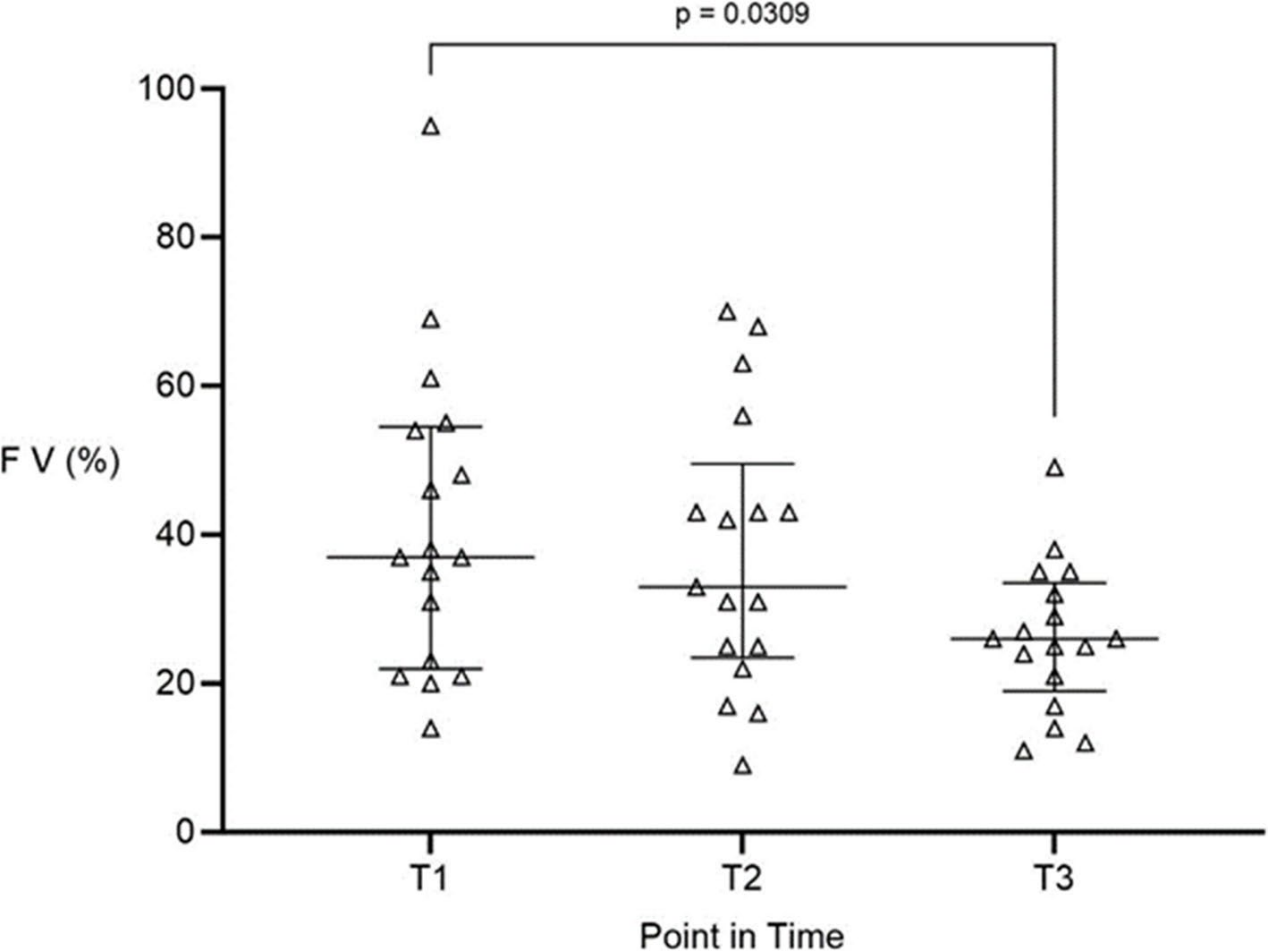
Intraoperative activity of coagulation factor V (FV) during liver transplantation. FV activity (%) measured at three intraoperative time points: T1 induction of general anesthesia, T2 end of anhepatic phase, T3 end of surgery. Data are presented as median with interquartile range. FV activity decreased progressively and significantly throughout the procedure

At baseline (T1), factor V activity was already markedly reduced and continued to decline throughout the intraoperative period (Fig. 1). Median FV activity levels decreased from 37% (22/55) at T1 to 33% (24/50) at T2, and further to 26% (19/34) at T3, with a statistically significant reduction between T1 and T3 (*p* = 0.0309).

**Fig. 2:**
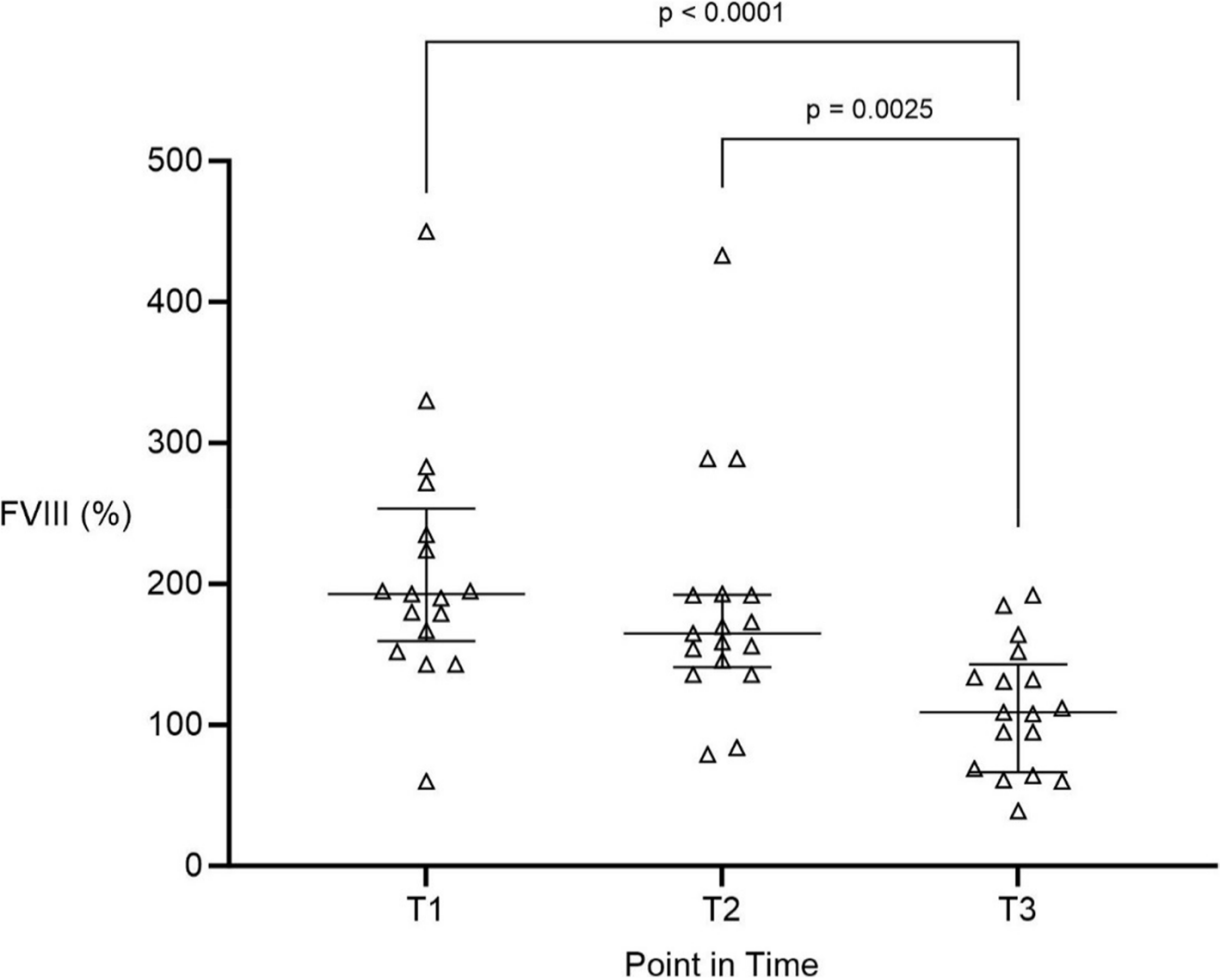
Intraoperative activity of coagulation factor VIII (FVIII) during liver transplantation. FVIII activity (%) measured at three intraoperative time points: T1 induction of general anesthesia, T2 end of anhepatic phase, T3 end of surgery. Data are presented as median with interquartile range. FVIII activity declined from supranormal to near-normal levels over the course of surgery

Factor VIII activity was supranormal at T1 and T2 but declined progressively during surgery (Fig. 2). Median values decreased from 193% (160/254) at T1 to 165% (141/193) at T2, reaching 109% (67/143) at T3, within the normal reference range. The decline was statistically significant between both T1 and T3 (*p* < 0.0001) and between T2 and T3 (*p* = 0.0025), indicating normalization of FVIII activity over the operative course.

**Fig. 3:**
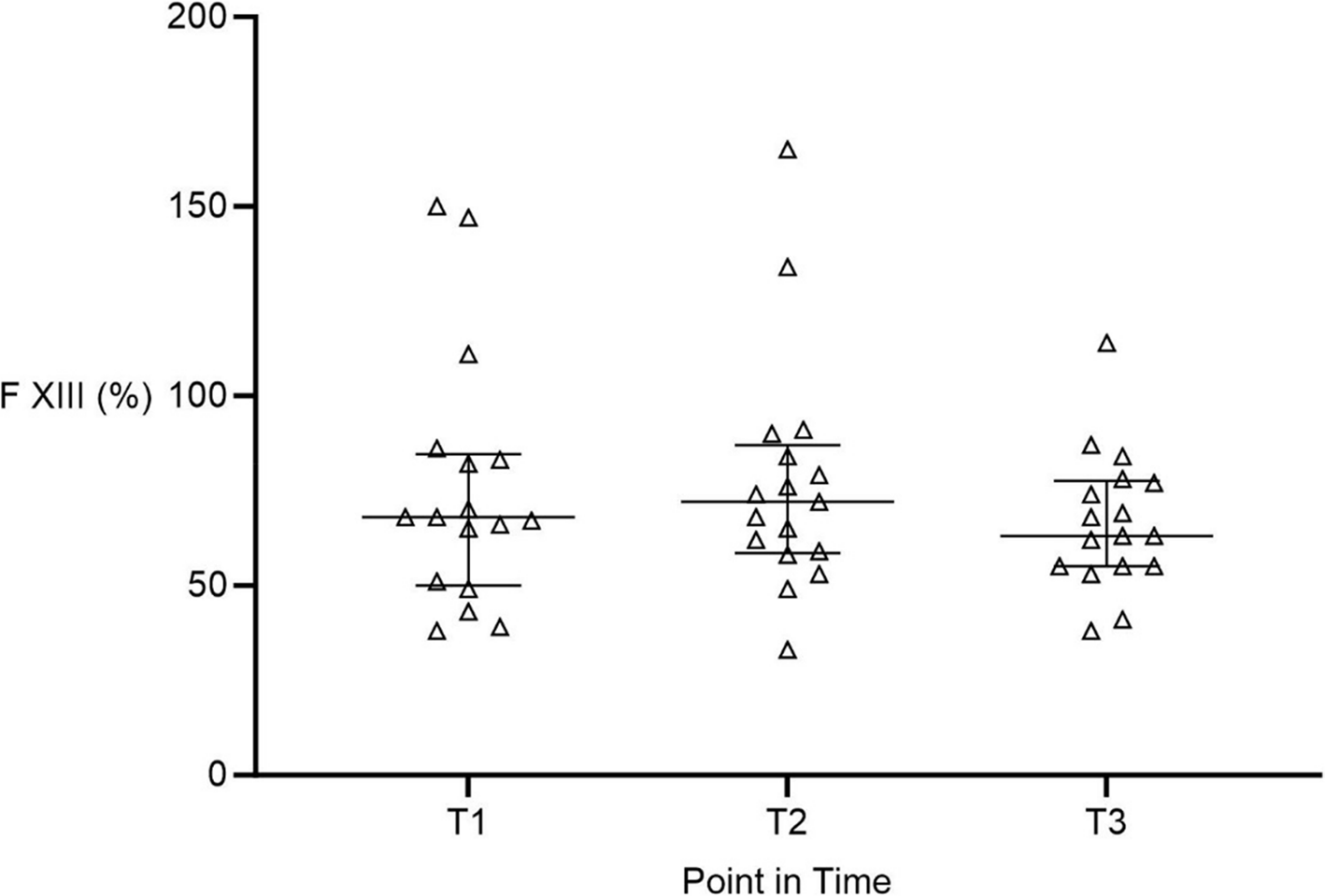
Intraoperative activity of coagulation factor XIII (FXIII) during liver transplantation. FXIII activity (%) measured at three intraoperative time points: T1 induction of general anesthesia, T2 end of anhepatic phase, T3 end of surgery. Data are presented as median with interquartile range. FXIII activity remained near the lower limit of normal with a slight, non-significant decrease during surgery

Factor XIII activity remained near the lower limit of normal at T1 and T2, with a slight, non-significant decrease at T3 (T1: 68% (50/85); T2: 72% (59/87); T3: 63% (55/78); Fig. 3).

To explore the potential mechanisms underlying the observed changes, correlation analyses were performed. No significant relationship was found between baseline FXIII activity and initial platelet count ( ρ = 0.374) as well as baseline FXIII activity and blood loss ( ρ = −0.4412). Likewise, intraoperative blood loss showed no significant correlation with changes in factor activity between T1 and T3 (blood loss vs FV: *ρ * = −0.442; blood loss vs FVIII: *ρ * = −0.3522; blood loss vs FXIII:  ρ = −0.4104).

## Discussion

This prospective analysis of 17 liver transplant recipients characterized the phase-specific intraoperative dynamics of coagulation factors V, VIII, and XIII. The study revealed distinct patterns: FV was markedly reduced at baseline and declined further during surgery, FVIII was supranormal at the start and gradually normalized, and FXIII remained near the lower limit of normal with only minor intraoperative changes. These findings provide a detailed, phase-specific profile of factor dynamics, offering insights into hemostatic modulation during liver transplantation.

While previous studies have characterized coagulation factor behavior in transfusion-free liver transplantation settings (Rengeiné et al. 2020), such cases represent a minority of clinical practice. In contrast, the present study focused on transfusion-supported, yet factor-free procedures, thereby reflecting real-world intraoperative conditions in which blood product administration and hemodilution interplay with hepatic synthetic dynamics. This perspective is essential for translating pathophysiologic findings into practical hemostatic management.

Liver transplantation is associated with substantial hemostatic alterations (Hartmann et al. 2016; Rengeiné et al. 2020; Lisman et al. 2002; Lewis et al. 1989). In patients with advanced liver disease these changes are increasingly interpreted within the framework of a rebalanced hemostasis, extensively described by the group of T. Lisman. In this model, concomitant decrease in pro- and anticoagulant pathways creates a fragile but often functionally preserved hemostatic equilibrium rather than a simple bleeding diathesis (Lisman and Porte 2010). Notably, thrombin-generating capacity has been shown to remain relatively preserved despite abnormal conventional coagulation tests (Tripodi et al. 2005; Lebreton et al. 2020). The phase-specific factor trajectories examined in our study provide additional clinical context to this concept. Although all three coagulation factors are synthesized in the liver, their activity is differentially affected by hepatic dysfunction. Baseline activities primarily reflect pre-existing liver dysfunction. FV, predominantly synthesized by hepatocytes, was already low at T1, underscoring its strong dependence on hepatic synthetic capacity (Rosing and Tans 1997; Duga et al. 2004). In contrast, FVIII, mainly produced by sinusoidal endothelial cells of the liver instead of hepatocytes and stabilized by elevated von Willebrand factor in cirrhosis, presented with supranormal levels. In addition, elevated FVIII levels in cirrhosis have been related to the presence and severity of portal hypertension and are thought to reflect endothelial activation and a prothrombotic shift in the portal circulation. Although not specifically investigated in the present study, this mechanism may represent a further potential explanation for the supranormal FVIII levels observed at baseline (Wu et al. 2024). FXIII, a heterotetramer with subunits derived from liver and extrahepatic sources (monocytes, macrophages, megakaryocytes), showed relative preservation, possibly due to compensatory synthesis and its long half-life (approximately 9 days) (Muszbek et al. 2011; Himmelreich et al. 1993); acquired partial reduction of FXIII has been described in severe liver disease (Rengeiné et al. 2020; 1978), however, the extrahepatic sources may partly mitigate the impact of hepatic injury on plasma FXIII activity. Comorbidities such as arterial hypertension, diabetes mellitus or nonalcoholic steatohepatitis (NASH) may also influence the systemic inflammatory status including FXIII. Unlike other coagulation factors like fibrinogen, FXIII has been reported to remain low during the first 48 h after transplantation (Rengeiné et al. 2020), suggesting it might be less suitable as a marker of early graft (dys-) function. These differential patterns may explain the distinct intraoperative trajectories observed. Although FXIII synthesis partly depends on megakaryocytes, no correlation between platelet count and FXIII activity was observed in this study, as opposed to prior data (Tacke et al. 2006). This may also reflect compensatory synthesis by monocytes or macrophages and the relatively long plasma half-life of FXIII, which might help maintain functional levels despite thrombocytopenia or impaired hepatic thrombopoietin production (Henry and Northup 2017; Kajihara et al. 2007; Pradella et al. 2011; Intagliata et al. 2019; Eto and Kunishima 2016; 2024).

During surgery, additional factors including cessation of hepatic synthesis during the anhepatic phase, dilution, consumption, and hyperfibrinolysis may contribute to further declines (Thaler et al. 2025; Lewis et al. 1989; Clevenger and Mallett 2014; Brezeanu et al. 2020). FV showed the most pronounced decrease, consistent with its short half-life (approximately 12 h) and sensitivity to synthetic disruption, highlighting its potential as an early marker for graft function; with the onset of functional graft activity, a prompt rise in FV levels can typically be observed indicating the restoration of hepatic synthesis capacity. This aligns well with prior studies highlighting the prognostic relevance of FV in liver transplantation (Lisman et al. 2002; Gorgen et al. 2019; Zulian et al. 2015). Low postoperative FV levels, especially below a threshold of 36%, have been demonstrated to predict early allograft dysfunction and graft loss within months following transplantation (Clevenger and Mallett 2014). In contrast, FVIII and FXIII appeared more resilient, likely due to extrahepatic production and kinetic stability, although FVIII declined more markedly than FXIII (Muszbek et al. 2011; 2024; Lison et al. 2011). The absence of a correlation between factor activities and intraoperative blood loss underscores the multifactorial nature of coagulation changes in this setting. This observation is consistent with previous work from the group of Lisman et al. given the poor performance of conventional coagulation parameters to reflect the overall hemostatic capacity in patients with liver disease, as simultaneous changes in pro- and anticoagulant pathways are not captured by routine assays (Lisman 2023). One additional player in this complexity may be hyperfibrinolysis, a well-recognized phenomenon during liver transplantation as repeatedly demonstrated in prior studies (Muszbek et al. 2011; Himmelreich et al. 1993; Thaler et al. 2025; Lisman et al. 2002); however, there is high variability in fibrinolytic phenotypes depending on the etiology and severity of the underlying liver disease (Blasi et al. 2020). Excess plasmin, as observed in hyperfibrinolysis, can degrade not only fibrin but also several coagulation factors – particularly FV and FVIII – thereby further reducing their plasma levels (Muszbek et al. 2011; Himmelreich et al. 1993; Lewis et al. 1989). This mechanism may serve as an additional explanation for the intraoperative decreases found in this study associated with surgical phases specifically prone to that coagulopathy like the anhepatic phase exhibiting pronounced factor changes. Early detection and timely treatment of clinically relevant hyperfibrinolysis might therefore contribute to stabilizing coagulation factor levels and overall hemostatic balance during transplantation.

The findings support a nuanced, factor-specific approach to coagulation management in liver transplantation. Such an individualized approach is in line with previous studies emphasizing that many bleeding events in liver disease are not solely caused by impaired hemostasis and that the optimal strategy for pro-hemostatic therapy remains uncertain. Consequently, current concepts advocate careful evaluation of bleeding etiology and the use of targeted interventions rather than routine correction of laboratory abnormalities. (Lisman et al. 2018; Lisman et al. 2022). FV may serve as an early indicator of hepatic synthetic recovery (even with hemostatic interventions like FFP administration), whereas the trajectories of FVIII and FXIII suggest that routine replacement may be unnecessary in the absence of clinical bleeding. Understanding these intraoperative patterns can help optimize individualized transfusion and substitution strategies, potentially reducing exposure to plasma products and associated risks.

This study uniquely provides high-resolution, phase-specific intraoperative data on FV, FVIII, and FXIII in a cohort without confounding by exogenous factor administration. Importantly, these findings extend previous observations from transfusion-free liver transplantation cohorts by demonstrating comparable yet distinct trajectories under clinically realistic, transfusion-supported conditions. This distinction is critical, as most liver transplantations involve blood product administration, which may itself modulate coagulation dynamics. By quantifying intraoperative factor kinetics in this context at three key surgical phases, the present study complements existing literature and highlights differential factor kinetics that may inform clinical decision-making.

This study holds some limitations to be acknowledged. First, the study was not powered to comprehensively assess factor kinetics nor were additional relevant variables measured that could have provided deeper insight into the underlying pathophysiology of coagulation factor dynamics, e.g. von Willebrand factor antigen (vWF-Ag); however, the study still provides valuable insight into the real-world peri-transplant course of coagulation factors. The paucity of data regarding graft characteristics and perioperative events such as reperfusion syndrome represents an important limitation as these factors may influence the observed variations in the synthesis of coagulation factors, especially FXIII which plays a key role in the interplay between inflammation and hemostasis; future studies should specifically address these aspects to allow for a robust evaluation of their independent effects on coagulation factor dynamics. Subgroup analyses according to liver failure severity or etiology were not feasible due to sample size constraints. Second, clinical endpoints such as bleeding complications and the postoperative recovery of factor activity were not evaluated. Last, the study cohort was small and highly selected, limiting generalizability.

## Conclusion

Conclusively, this study demonstrates distinct phase-specific intraoperative dynamics of FV, FVIII, and FXIII during liver transplantation. FV is highly sensitive to hepatic dysfunction and intraoperative stress, FVIII declines from supranormal baseline levels, and FXIII remains relatively stable. Recognizing these differential patterns may support targeted, factor-specific management and improved hemostatic monitoring in liver transplant recipients.

## Data Availability

All data generated or analyzed during this study are included in this published article.

## Abbreviations

aPTT: Activated Partial Thromboplastin Time
BMI: Body Mass Index
FFP: Fresh Frozen Plasma
FV: Coagulation factor V
FVIII: Coagulation factor VIII
FXIII: Coagulation factor XIII
HCC: Hepatocellular carcinoma
INR: International Normalized Ratio
IQR: Interquartile Range
MELD: Model for End-stage Liver Disease
NASH: Nonalcoholic Steatohepatitis
vWF-Ag: Von Willebrand factor antigen

## References

[CR1] Biland L, Duckert F, Prisender S, Nyman D. Quantitative estimation of coagulation factors in liver disease. The diagnostic and prognostic value of factor XIII, factor V and plasminogen. Thromb Haemost. 1978;39(3):646–56 (PMID: 705694). 705694

[CR2] Blasi A, Patel VC, Adelmeijer J, Azarian S, Hernandez Tejero M, Calvo A, et al. Mixed fibrinolytic phenotypes in decompensated cirrhosis and acute-on-chronic liver failure with hypofibrinolysis in those with complications and poor survival. Hepatology. 2020;71(4):1381–90. 10.1002/hep.30915. (Epub 2019 Oct 24. PMID: 31465557; PMCID: PMC7187291.). 31465557 10.1002/hep.30915 PMC7187291

[CR3] Brezeanu LN, Brezeanu RC, Diculescu M, Droc G. Anaesthesia for liver transplantation: an update. J Crit Care Med. 2020;6(2):91–100. 10.2478/jccm-2020-0011. (PMID: 32426515; PMCID: PMC7216023). 10.2478/jccm-2020-0011 PMC7216023 32426515

[CR4] Clevenger B, Mallett SV. Transfusion and coagulation management in liver transplantation. World J Gastroenterol. 2014;20(20):6146–58. 10.3748/wjg.v20.i20.6146. (PMID: 24876736; PMCID: PMC4033453). 24876736 10.3748/wjg.v20.i20.6146 PMC4033453

[CR5] Duga S, Asselta R, Tenchini ML. Coagulation factor V. Int J Biochem Cell Biol. 2004;36(8):1393–9. 10.1016/j.biocel.2003.08.002. (PMID: 15147718). 15147718 10.1016/j.biocel.2003.08.002

[CR6] Eto K, Kunishima S. Linkage between the mechanisms of thrombocytopenia and thrombopoiesis. Blood. 2016;127(10):1234–41. 10.1182/blood-2015-07-607903. (Epub 2016 Jan 19. PMID: 26787737; PMCID: PMC4786834.). 26787737 10.1182/blood-2015-07-607903 PMC4786834

[CR7] Forkin KT, Colquhoun DA, Nemergut EC, Huffmyer JL. The coagulation profile of end-stage liver disease and considerations for intraoperative management. Anesth Analg. 2018;126(1):46–61. 10.1213/ANE.0000000000002394. (PMID: 28795966). 28795966 10.1213/ANE.0000000000002394

[CR8] Gorgen A, Prediger C, Prediger JE, Chedid MF, Backes AN, de Araujo A, et al. Serum factor V is a continuous biomarker of graft dysfunction and a predictor of graft loss after liver transplantation. Transplantation. 2019;103(5):944–51. 10.1097/TP.0000000000002429. (PMID: 30130328). 30130328 10.1097/TP.0000000000002429

[CR9] Hartmann M, Szalai C, Saner FH. Hemostasis in liver transplantation: pathophysiology, monitoring, and treatment. World J Gastroenterol. 2016;22(4):1541–50. 10.3748/wjg.v22.i4.1541. (PMID: 26819521; PMCID: PMC4721987). 26819521 10.3748/wjg.v22.i4.1541 PMC4721987

[CR10] Henry Z, Northup PG. The rebalanced hemostasis system in end-stage liver disease and its impact on liver transplantation. Int Anesthesiol Clin. 2017;55(2):107–20. 10.1097/AIA.0000000000000139. (PMID: 28221169). 28221169 10.1097/AIA.0000000000000139

[CR11] Himmelreich G, Müller C, Isenberg C, Bechstein WO, Roissant R, Riess H. Factor XIII activity during orthotopic liver transplantation. Semin Thromb Hemost. 1993;19(3):243–5. 10.1055/s-2007-994033. (PMID: 7689750). 7689750 10.1055/s-2007-994033

[CR12] Hollestelle MJ, Thinnes T, Crain K, Stiko A, Kruijt JK, van Berkel TJ, et al. Tissue distribution of factor VIII gene expression in vivo–a closer look. Thromb Haemost. 2001;86(3):855–61 (PMID: 11583319). 11583319

[CR13] Hollestelle MJ, Geertzen HG, Straatsburg IH, van Gulik TM, van Mourik JA. Factor VIII expression in liver disease. Thromb Haemost. 2004;91(2):267–75. 10.1160/TH03-05-0310. (PMID: 14961153). 14961153 10.1160/TH03-05-0310

[CR14] Intagliata NM, Davis JPE, Lafond J, Erdbruegger U, Greenberg CS, Northup PG, et al. Acute kidney injury is associated with low factor XIII in decompensated cirrhosis. Dig Liver Dis. 2019;51(10):1409–15. 10.1016/j.dld.2019.03.011. (PMID: 30967339). 30967339 10.1016/j.dld.2019.03.011

[CR15] Kajihara M, Okazaki Y, Kato S, Ishii H, Kawakami Y, Ikeda Y, et al. Evaluation of platelet kinetics in patients with liver cirrhosis: similarity to idiopathic thrombocytopenic purpura. J Gastroenterol Hepatol. 2007;22(1):112–8. 10.1111/j.1440-1746.2006.04359.x. (PMID: 17201890). 17201890 10.1111/j.1440-1746.2006.04359.x

[CR16] Kozek-Langenecker S, Sørensen B, Hess JR, Spahn DR. Clinical effectiveness of fresh frozen plasma compared with fibrinogen concentrate: a systematic review. Crit Care. 2011;15(5):R239. 10.1186/cc10488. (Epub 2011 Oct 14. PMID: 21999308; PMCID: PMC3334790). 21999308 10.1186/cc10488 PMC3334790

[CR17] Lebreton A, Sinegre T, Lecompte T, Talon L, Abergel A, Lisman T. Thrombin generation and cirrhosis: state of the art and perspectives. Semin Thromb Hemost. 2020;46(6):693–703. 10.1055/s-0040-1715102. (Epub 2020 Aug 20. PMID: 32820480). 32820480 10.1055/s-0040-1715102

[CR18] Lewis JH, Bontempo FA, Awad SA, Kang YG, Kiss JE, Ragni MV, et al. Liver transplantation: intraoperative changes in coagulation factors in 100 first transplants. Hepatology. 1989;9(5):710–4. 10.1002/hep.1840090509. (PMID: 2651269; PMCID: PMC3032392). 2651269 10.1002/hep.1840090509 PMC3032392

[CR19] Lisman T. How to assess hemostasis in patients with severe liver disease. Hematology Am Soc Hematol Educ Program. 2023;2023(1):267–73. 10.1182/hematology.2023000479. (PMID: 38066858; PMCID: PMC10727047). 38066858 10.1182/hematology.2023000479 PMC10727047

[CR20] Lisman T, Porte RJ. Rebalanced hemostasis in patients with liver disease: evidence and clinical consequences. Blood. 2010;116(6):878–85. 10.1182/blood-2010-02-261891. (Epub 2010 Apr 16. PMID: 20400681). 20400681 10.1182/blood-2010-02-261891

[CR21] Lisman T, Leebeek FW, de Groot PG. Haemostatic abnormalities in patients with liver disease. J Hepatol. 2002;37(2):280–7. 10.1016/s0168-8278(02)00199-x. (PMID: 12127437). 12127437 10.1016/s0168-8278(02)00199-x

[CR22] Lisman T, Kleiss S, Patel VC, Fisher C, Adelmeijer J, Bos S, et al. In vitro efficacy of pro- and anticoagulant strategies in compensated and acutely ill patients with cirrhosis. Liver Int. 2018;38(11):1988–96. 10.1111/liv.13882. (Epub 2018 May 30. PMID: 29768734; PMCID: PMC6220788). 29768734 10.1111/liv.13882 PMC6220788

[CR23] Lisman T, Caldwell SH, Intagliata NM. Haemostatic alterations and management of haemostasis in patients with cirrhosis. J Hepatol. 2022;76(6):1291–305. 10.1016/j.jhep.2021.11.004. (PMID: 35589251). 35589251 10.1016/j.jhep.2021.11.004

[CR24] Lison S, Weiss G, Spannagl M, Heindl B. Postoperative changes in procoagulant factors after major surgery. Blood Coagul Fibrinolysis. 2011;22(3):190–6. 10.1097/MBC.0b013e328343f7be. (PMID: 21245747). 21245747 10.1097/MBC.0b013e328343f7be

[CR25] Mangla A, Hamad H, Killeen RB, Kumar A. Factor XIII Deficiency. 2024 Feb 12. In: StatPearls. Treasure Island (FL): StatPearls Publishing; 2025 Jan–. PMID: 32491399. 32491399

[CR26] Muszbek L, Bereczky Z, Bagoly Z, Komáromi I, Katona É. Factor XIII: a coagulation factor with multiple plasmatic and cellular functions. Physiol Rev. 2011;91(3):931–72. 10.1152/physrev.00016.2010. (PMID: 21742792). 21742792 10.1152/physrev.00016.2010

[CR27] Northup PG, Sundaram V, Fallon MB, Reddy KR, Balogun RA, Sanyal AJ, et al. Hypercoagulation and thrombophilia in liver disease. J Thromb Haemost. 2008;6(1):2–9. 10.1111/j.1538-7836.2007.02772.x. (Epub 2007 Sep 24. PMID: 17892532). 17892532 10.1111/j.1538-7836.2007.02772.x

[CR28] Pradella P, Bonetto S, Turchetto S, Uxa L, Comar C, Zorat F, et al. Platelet production and destruction in liver cirrhosis. J Hepatol. 2011;54(5):894–900. 10.1016/j.jhep.2010.08.018. (PMID: 21145808). 21145808 10.1016/j.jhep.2010.08.018

[CR29] Rassi AB, d’Amico EA, Tripodi A, da Rocha TRF, Migita BY, Ferreira CM, et al. Fresh frozen plasma transfusion in patients with cirrhosis and coagulopathy: effect on conventional coagulation tests and thrombomodulin-modified thrombin generation. J Hepatol. 2020;72(1):85–94. 10.1016/j.jhep.2019.09.008. (Epub 2019 Sep 16. PMID: 31536747). 31536747 10.1016/j.jhep.2019.09.008

[CR30] Rengeiné TK, Máthé Z, Piros L, Dinya E, Smudla A, Mándli T, et al. How much is the inevitable loss of different coagulation factors during blood product-free liver transplantations? Transplant Proc. 2020;52(10):2988–95. 10.1016/j.transproceed.2020.05.006. (Epub 2020 Jul 9. PMID: 32653159). 32653159 10.1016/j.transproceed.2020.05.006

[CR31] Rosing J, Tans G. Factor V. Int J Biochem Cell Biol. 1997;29(10):1123–6. 10.1016/s1357-2725(97)00040-x. (PMID: 9438374). 9438374 10.1016/s1357-2725(97)00040-x

[CR32] Sinegre T, Duron C, Lecompte T, Pereira B, Massoulier S, Lamblin G, et al. Increased factor VIII plays a significant role in plasma hypercoagulability phenotype of patients with cirrhosis. J Thromb Haemost. 2018;16(6):1132–40. 10.1111/jth.14011. (Epub 2018 May 17. PMID: 29577605). 29577605 10.1111/jth.14011

[CR33] Tacke F, Fiedler K, von Depka M, Luedde T, Hecker H, Manns MP, et al. Clinical and prognostic role of plasma coagulation factor XIII activity for bleeding disorders and 6-year survival in patients with chronic liver disease. Liver Int. 2006;26(2):173–81. 10.1111/j.1478-3231.2005.01205.x. (PMID: 16448455). 16448455 10.1111/j.1478-3231.2005.01205.x

[CR34] Thai C, Oben C, Wagener G. Coagulation, hemostasis, and transfusion during liver transplantation. Best Pract Res Clin Anaesthesiol. 2020;34(1):79–87. 10.1016/j.bpa.2020.03.002. (Epub 2020 Mar 16. PMID: 32334789). 32334789 10.1016/j.bpa.2020.03.002

[CR35] Thaler S, Zorn A, Aster I, Koliogiannis D, Renz BW, Guba M, et al. Hyperfibrinolysis detection during liver transplantation using viscoelastometry. Clin Transplant. 2025;39(5):e70179. 10.1111/ctr.70179. (PMID: 40349145; PMCID: PMC12066000). 40349145 10.1111/ctr.70179 PMC12066000

[CR36] Tripodi A, Mannucci PM. The coagulopathy of chronic liver disease. N Engl J Med. 2011;365(2):147–56. 10.1056/NEJMra1011170. (PMID: 21751907). 21751907 10.1056/NEJMra1011170

[CR37] Tripodi A, Salerno F, Chantarangkul V, Clerici M, Cazzaniga M, Primignani M, et al. Evidence of normal thrombin generation in cirrhosis despite abnormal conventional coagulation tests. Hepatology. 2005;41(3):553–8. 10.1002/hep.20569. (PMID: 15726661). 15726661 10.1002/hep.20569

[CR38] Tripodi A, Primignani M, Mannucci PM. Abnormalities of hemostasis and bleeding in chronic liver disease: the paradigm is challenged. Intern Emerg Med. 2010;5(1):7–12. 10.1007/s11739-009-0302-z. (Epub 2009 Aug 28. PMID: 19714443). 19714443 10.1007/s11739-009-0302-z

[CR39] Wu Z, Xiao Y, Qi Z, Guo T, Tong H, Wang Y. Effect of factor VIII and FVIII/PC ratio on portal vein thrombosis in liver cirrhosis: a systematic review and meta-analysis. BMC Gastroenterol. 2024;24(1):320. 10.1186/s12876-024-03399-1. (PMID: 39300356; PMCID: PMC11411769). 39300356 10.1186/s12876-024-03399-1 PMC11411769

[CR40] Zulian MC, Chedid MF, Chedid AD, Grezzana Filho TJ, Leipnitz I, de Araujo A, et al. Low serum factor V level: early predictor of allograft failure and death following liver transplantation. Langenbecks Arch Surg. 2015;400(5):589–97. 10.1007/s00423-015-1290-2. (PMID: 25708642). 25708642 10.1007/s00423-015-1290-2

